# Report from the Medical Library Association’s InSight Initiative Summit 1: Engaging Users in a Disruptive Era

**DOI:** 10.5195/jmla.2018.561

**Published:** 2018-10-01

**Authors:** Katherine G. Akers

**Affiliations:** Editor-in-Chief, *Journal of the Medical Library Association*, and Biomedical Research and Data Specialist, Shiffman Medical Library, Wayne State University, Detroit, MI

## Abstract

At the Medical Library Association’s Insight Initiative Summit 1, held March 6–7, 2018, academic and hospital librarians and publishing industry partners came together to discuss their shared role in engaging users of health sciences information in an era in which “disruptors” such as pirate websites, scientific collaboration networks, and preprint servers pose threats to traditional means of access to scholarly content. Through a mixture of keynote talks, themed panel discussions, and small-group problem-solving exercises, the summit program raised important questions, sparked conversation, and provided insight into the need for both libraries and publishing organizations to improve their user experience, lower their barriers to access, and offer value to users that cannot be provided by competitors, including helping authors and students become informed, responsible advocates for and consumers of scholarly publications. The key takeaways from the summit are expected to impact libraries’ and publishers’ strategies and stimulate the cocreation of enduring materials to enhance user engagement in disseminating and discovering scientific and medical information.

The Medical Library Association (MLA) InSight Initiative Summit 1, held March 6–7, 2018, in Chicago, IL, brought together library leaders and publishing industry partners to engage in high-level, high-value dialogue on issues of common interest that impact the health information profession. The theme of this summit, “Engaging Users in a Disruptive Era,” addressed the following topics:

- leveraging specialized discovery tools to maximize user engagement
- security and the future of Internet protocol (IP) address authentication
- lessons learned from pirate sites
- imagining the ideal social networking site for collaboration and sharing

The program included a mixture of keynote talks, themed panel discussions, and small-group problem-solving exercises.

## WELCOME

Members of the MLA InSight Initiative Task Force welcomed summit participants and thanked Dan Doody and Rich Lampert from Doody Consulting and other members of the task force and Insight Summit 1 Program Committee for organizing the summit, the Association of Academic Health Sciences Libraries (AAHSL) and Elsevier for financial contributions, and representatives of publishing organizations for engaging with librarians. Participants were encouraged to extend the summit conversation online using the Twitter hashtag #mlainsight.

## SMALL GROUP EXERCISE #1

Participants became acquainted with each other by sharing their names, places of residence, professional affiliations and roles, and what they hoped to get out of the summit.

## KEYNOTE #1: ENGAGING USERS IN AN ERA OF COMMODITIZATION

### William Garrity, Deputy University Librarian and Chief of Staff, University of California–Davis

William Garrity discussed the importance of academic libraries providing experiences that appeal to users and help them achieve their objectives. He stated that much of what librarians do will become or has already become commoditized. That is, today’s users increasingly procure services or goods based overwhelmingly on convenience and in a manner that is disconnected from intermediaries. If libraries cannot offer appealing user experiences with added value that is not provided by competitors, they risk becoming irrelevant to users, who risk being ill-served as a consequence.

Garrity commenced his keynote talk by providing examples of web-based services that have disrupted traditional paths of service provision, such as the Leafly app for obtaining access to medical marijuana, Teleradiology Solutions for outsourcing the reading of medical imaging, the Uber ride-sharing app, and the online retailer Amazon. Asked the question “what qualities do these share?,” audience members answered “convenience,” “ease of use,” “always present,” “immediate outcomes,” “no geographic constraints,” “access to unlimited inventory or resources,” and “ability to complete transactions without speaking to a person.” Garrity said that libraries should strive to provide services and access to information resources in a manner that co-opts these essential qualities.

Garrity believes that medical libraries are well-positioned to thrive in these uncertain times. Users of medical libraries constitute a monolithic group with common objectives. They are focused; they want to get research done and help patients. They are respectful of expertise, seeing librarians as partners in clinical, educational, and research activities. Medical librarians are particularly innovative, nimble, enabling of users, quick to act, and supportive of faculty. Thus, they are in a good position to help users have positive experiences and succeed.

Garrity shared a three-point strategy for dealing with challenges faced by libraries: (1) make evidence-based decisions, (2) adopt orthogonal perspectives (i.e., approach problems from a nonlibrary point-of-view), and (3) do things at scale. As an example, Garrity’s main library needed renovation. Although faculty believed that they were the primary library clientele, librarians performed a year-long study of library users to obtain evidence. They found that 93% of library visitors were students and less than 3% were faculty. Of the student visitors, less than 1/3 engaged with library collections, services, or expertise during their visits; most simply used the library as a space for studying. These findings allowed librarians to “reset the campus narrative about the library” and aim renovations at creating an academic center for improving student success. To address the issue of scale, librarians are inserting learning objects into normal learning and research workflows, as there are not enough librarians to teach information literacy (IL) to all students.

During their study, the librarians also found that students often studied in cramped spaces, sometimes even sitting on the floor, like “O’Hare on the worst travel day.” Garrity suggested that insight into the design of library space could come from the environments in which students will work after graduation. For example, modern start-ups and media firms occupy open environments that contain moveable furniture and foster collaboration. Thus, libraries could create student spaces in collaboration with companies like WeWork, which designs and leases coworking spaces that are aesthetically pleasing and flexibly meet user needs.

Garrity noted several issues that could drive users away from the library as a source for information. Most library users are “satisficers” who do not need much data to make a decision, whereas librarians tend to be “maximizers”; users might want only a few articles to get started instead of hundreds of references. People are outcome-focused; their objective is simply to get work done. Information may be indistinguishable to users: A student may want *a* journal article instead of *the* journal article, and how they get it is irrelevant. People have more choices for sourcing information, with even Garrity admitting that he used Google Scholar rather than his library’s search tool for researching his talk. Library tools and services are often invisible, causing users to lose connection with librarians’ expertise. Finally, library tools and services are too difficult to use: “If any business put their customers through what we put our users through, they’d be out of business.”

As an example of how libraries can attract users, Garrity spoke about his library’s new Archives and Institutional Assets program. His university has recently seen a burgeoning of research programs, resulting in more researchers experiencing the “headache” of complying with open access and data management mandates. At the same time, given the digital medium of today’s research process, a traditional university archives approach struggled to capture the story of campus growth. To meet multiple needs at once, Garrity’s library has developed a program that simultaneously supports researchers’ needs from the beginning of their careers and that preserves and provides access to university materials of enduring value. Together, his library’s Archives and Institutional Assets, Scholarly Communications, and Data Management programs rely on referrals among liaison and specialist librarians to connect researchers with library staff who can alleviate their “headaches,” while also demonstrating the impact of their research.

In conclusion, Garrity proposed a three-point plan for librarian action in an era of commoditization: (1) Focus on making users’ information experiences positive. Librarians should put themselves in the role of a new student or faculty member who tries to use library tools and services; “you might be appalled at what we expect people to do.” Librarians should ask themselves: Are our tools intuitive to use? Are they convenient (i.e., low-to-no barriers)? Are we enabling our users’ success? (2) Do we strive to offer value that is not otherwise available from competitors? (3) Do we make expertise visible, relevant, and intrinsic to the user experience?

### Question-and-answer session

The main theme of audience responses was the idea of the library as a physical place for collaboration. One participant emphasized that librarians “cannot view libraries as a temple of librarians.” Rather, libraries should be “true interprofessional educational homes,” and user communities should be invited into these spaces to collaboratively solve problems. However, a librarian who said he had long worked to advance interprofessional education expressed concern that different user communities are monolithic entities who engage in tribal affiliation, saying that “forced mixed groups fall apart.” This librarian also expressed interest in applying diversity practices (e.g., advocacy, allyship, critical librarianship) and design thinking processes to the redevelopment of library spaces and services as well as working with industry partners toward this end. Garrity agreed with the importance of creating library spaces for diversity and inclusion. He described how the consulting firm brightspot discovered that students at his institution would rather learn from each other than from librarians. Based on this evidence, library spaces were dedicated to specific student advocacy groups, into which librarians “dropped in” when students were studying together and were poised to learn. This experience helped librarians break out of the “I am the informational professional” perspective and meet students where they are.

Garrity ended the session by reminding the audience that librarians are service oriented and nonthreatening. They have history of bringing people together to work on things and are perceived to be ecumenical problem-solvers rather than empire-builders. Thus, they are well positioned to foster relationships across campus.

## PANEL #1: SUCCESS STORIES IN USER ENGAGEMENT

The objective of this panel was to show how valued content, ease of access, and ease of use combine to create appealing user experiences. Participants were expected to learn about different approaches to drawing in users and retaining their loyalty, which could serve as components of a “tool kit” to improve the content and functionality of information resources.

### What ResearchGate gets right! (Lessons for libraries and publishers?)

#### Lisa Janicke Hinchliffe, Coordinator for Information Literacy Services and Instruction, University Library, University of Illinois–Urbana-Champaign

Contrary to the low opinion of ResearchGate often held by librarians and publishers, Lisa Janicke Hinchliffe proposed that ResearchGate “gets a lot of things right” and encouraged libraries and publishers to consider adopting some aspects of ResearchGate that are most attractive to users. She prefaced her talk by pointing out that librarians have been worried about going out of business since the advent of microfilm and photocopiers and posited that although new technologies “can disrupt our world, they will not put us out of business.”

When Hinchliffe asked participants, “What is ResearchGate?,” responses included “Facebook for scientists,” “a social networking service for scientists,” “a discovery platform,” and “a place where I get free pdfs.”

ResearchGate allows the creation of user profiles, the formation of professional networks, private sharing and public downloading of publications, usage metrics (i.e., reads, citations), user updates about projects and works-in-progress, and the asking and answering of research-related questions within its user community. Although some consider ResearchGate a pirate site, it holds both licit and illicit content, with some studies reporting that less than half of its content infringes on copyright.

Currently, ResearchGate has 13 million users and is the ~260th most popular website in the world. It is considered *the* scholarly communication/collaboration network. ResearchGate’s mission is “to connect the world of science and make research open to all,” which Hinchliffe mentioned could also be libraries’ or publishers’ mission. However, there is clear competition between ResearchGate and libraries and publishers. ResearchGate is a for-profit company and has a lot of venture capital, against which it will be difficult for libraries to compete.

Hinchliffe highlighted several things that ResearchGate “gets right”:

- It has low-to-no barriers to entry. Users can search for, read, and download publications easily and for free.
- It is centered on the individual. Users can carry their profile from institution to institution, and the platform is extremely user-focused; using ResearchGate is all about “you!”
- It allows multiple modes of engagement. Users can choose how to use ResearchGate for their own purposes.
- It provides a positive and encouraging environment. Users can expect quick access to portable document format files (PDFs) and answers to questions. Users also frequently receive “Congratulations!” emails notifying them of personal achievements (and which draw them back into the site).
- Its value outweighs its annoyances.

In conclusion, Hinchliffe asked audience members to consider whether these characteristics are also exhibited by their organizations.

### Preprints in biology and medicine

#### John Inglis, Co-Founder, bioRxiv and medRxiv, and Executive Director, Cold Spring Harbor Laboratory Press, Cold Spring Harbor, NY

John Inglis described the growth of bioRxiv and the development of medRxiv, preprint servers for the life sciences and medicine, respectively. Inglis defined “preprint” as a complete but unpublished manuscript yet to be certified by peer review that is distributed by its author before or at the time of journal submission and “preprint server” as a dedicated, journal-independent platform for distributing preprints in a defined domain.

Launched in 2013 as a not-for-profit operation of Cold Spring Harbor Laboratory, bioRxiv is a preprint server for the life sciences that is modeled on the arXiv preprint server for physics and mathematics. After preprints are posted to bioRxiv by their authors, they are screened for appropriateness but are not peer-reviewed. Each preprint is placed a single subject category and is labeled as “new,” “confirmatory,” or “contradictory.” bioRxiv employs a lightweight submission system; makes preprints available twenty-four to forty-eight hours after submission with a date stamp, digital object identifier (DOI), and persistent uniform resource locator (URL); and displays usage metrics (e.g., downloads, views, altmetrics). bioRxiv is free for authors and readers and does not require user registration.

bioRxiv currently contains 22,000 manuscripts posted by 120,000 authors from 7,600 institutions in 104 countries. The most popular subject categories are neuroscience, bioinformatics, evolutionary biology, and genomics/genetics. The institutions most highly represented in bioRxiv read like an “A-team” of research institutions, with Stanford University, University of Cambridge, University of Oxford, University of Washington, and Harvard University at the top of the list. bioRxiv has grown rapidly since its launch, from 50 posts per month in 2013 to 1,200 posts per month in early 2018. This expansion of content is paralleled by an increase in usage: currently, 750,000 downloads and 1,750,000 abstract views per month.

Multiple lines of evidence indicate that readers actively engage with preprints in bioRxiv. For instance, 18% of preprints have Altmetric scores of 20 or above, and 9% have reader comments. bioRxiv preprints also receive a total of 115,000 tweets per year and are increasingly highlighted and discussed on online scientific communities such as MicroNow and preLights.

A valuable feature of bioRxiv is its integration with journal submission systems. Currently, 120 journals from 31 publishers allow authors to transfer their manuscript from bioRxiv to their submission systems with only “one click.” Also, 11 publishers allow authors to transfer a copy of their manuscript to bioRxiv upon journal submission.

The benefits of preprint servers to authors are numerous: They allow immediate sharing of research findings, may confer a citation advantage, provide evidence of productivity for early career researchers, ensure against “scooping,” and accelerate scientific progress. The success of preprint servers is evidenced by their quick proliferation, with approximately thirty preprint servers now covering nearly all scholarly disciplines, including agriculture, earth sciences, nutrition, psychology, social sciences, law, and the humanities.

A preprint server for medicine could also have several benefits, such as accelerating the sharing of new medical information, making less publishable outputs (e.g., protocols, technical reports, quality innovations) more available, and increasing the availability of clinical trial data. However, several concerns over the sharing of medical preprints pertain to harms to the public due to incorrect medical information (which can be magnified by the media), the persistence of outdated manuscripts that may have drastically changed as a result of peer review, the manipulation of medical information by commercial interests, and an undermining of efforts such as ClinicalTrials.gov. Such concerns are currently under vigorous public debate.

Amid these discussions, the medical preprint server medRxiv will launch later this year. Like bioRxiv, medRxiv is a not-for-profit initiative of Cold Spring Harbor Laboratory and founding partners and will be integrated with external journal submission and other manuscript assessment systems. However, medRxiv will employ more rigorous approaches to mitigate the risks of sharing the early results of medical research. It will have an advisory board composed of clinicians, journal editors, and other stakeholders. Submissions will be screened by qualified professionals using defined criteria such as the presence of author academic/professional affiliations, a conflict of interest statement, and documentation of institutional review board (IRB) approval or exemption. Preprints will feature disclaimers emphasizing the lack of peer review to alert the public, journalists, and health care professionals. Furthermore, like biorXiv, medRxiv will only allow preprints describing original research, excluding opinion pieces, reviews, and case reports.

### Resource access in the 21st century (RA21)

#### Heather Flanagan, Academic Pilot Coordinator, RA21

Heather Flanagan described Resource Access for the 21st Century (RA21), a new approach to user authentication that overcomes problems associated with Internet protocol (IP)–based authentication. RA21 is a joint initiative of the International Association of Scientific, Technical, and Medical Publishers (STM) and the National Information Standards Organization (NISO) that aims to facilitate a seamless user experience and preserve user privacy by optimizing access protocols across key stakeholder groups, including universities, libraries, information resource vendors, publishers, and identity federation operators.

A new approach to authentication is needed because users increasingly access scholarly content from off-campus networks. However, publisher and campus pathways for providing off-network access are complex, cumbersome, and insecure and have not kept pace with the positive user experience offered by other online services (e.g., Google, Facebook, LinkedIn). Despite the disadvantages of IP authentication, most off-campus solutions such as virtual private networks (VPNs)/proxy servers, device pairing, and Google Scholar’s Campus Activated Subscriber Access (CASA) still leverage institutional IP address recognition and require user setup in advance. Due to difficulty accessing content from publisher platforms, even when they are fully entitled to access, users are turning to alternative, often illegitimate sources of content (e.g., SciHub) due to their ease of use. Flanagan emphasized the need to develop an approach to authentication that is a better experience for users, quoting Steve Jobs: “You have to start with the customer experience and work your way back to technology.”

RA21 seeks to implement the same approach to authentication employed by consumer sites but with the added protection of user privacy. For example, a user can set up a Doodle account using their Google login information, but this results in Doodle having too much information about the user, including name, email address, and picture. With RA21, however, when users are off-campus, they authenticate through their institutions, and the publisher receives attributes about users (e.g., their roles within the institution) but not the users’ identities. Additional benefits of RA21 include users’ ability to start their searches from their tool of choice (e.g., Google, PubMed) instead of first needing to go through an institutional portal; the lack needing yet another account ID and password, as RA21 leverages a user’s existing institutional credentials; the ability to block a single user account instead of an IP address; and the ability for subscribers to receive more granular usage statistics.

As RA21 rolls out, all stakeholders must make changes. Content providers will need changes to their websites and services, librarians will have to answer user questions and work with central campus informational technology departments, and identity providers will need to build and strengthen trust. In conclusion, Flanagan presented the future roadmap for RA21, highlighting their outreach to and engagement with key stakeholder communities throughout 2018 and the passing of the project lead from STM to NISO for implementation after 2018.

### Question-and-answer session

The main theme of the post-panel discussion concerned the role of controlled access to resources in future decades, when most scholarly content is expected to be open access. A participant remarked that we will no longer need user authentication in an “open access world” and that we are pushing our users to become the products (e.g., monetization of user clicks in ResearchGate). Another participant questioned the future of journals, stating that “enlightened publishers are thinking of themselves as curators” who evaluate and point readers to the most important work; that is, “journals will attempt to do evaluation without all the baggage.” When asked how much consensus or debate over the future of journals exists in publisher circles, representatives of publishing organizations replied that “we talk about preprint servers all the time” and “we talk about the changing landscape of incentives.” At the end, a participant shared that she knew about journals that use ResearchGate as their publishing platform, with the editor telling authors to post their manuscripts on ResearchGate after their peer review as a mode of publication.

## SMALL GROUP EXERCISE #2

Participants gathered in small groups with roughly equal representation by librarians and publishers to discuss the topics of “lessons learned from pirate sites” and “security and the future of IP authentication.” The groups defined broad problems, identified areas of concern or controversy, and suggested next steps.

### Lessons learned from pirate sites

#### Questions to consider

- Which pirate sites are most popular among the population of potential library users?
- Which sites do IP owners see as particularly threatening to their businesses?
- If specific content is available both through the library and a pirate site, why should a rational user choose to access the content through the pirate site?
- What functionality exists on pirate sites that should be available on systems delivering licensed content? What are the barriers to accomplishing this?

#### Group 1

Group 1 defined a pirate site as proactively violating copyright (e.g., SciHub). Some members of our group also considered ResearchGate as a pirate site. We felt that users do not necessarily want to infringe on copyright, but they do not understand it. If we are considering only open access content, however, then there are no pirate sites. Thus, there is likely a finite lifespan for pirate sites because they will not add value when most or all content is open.

It is difficult to know which sites are most popular among users because some libraries block pirate sites, users are often outside of the institution’s IP range when they use these sites, usage statistics are not available, and users often do not tell the library that they use these sites.

There is a threat to both IP owners and libraries when users fail to recognize where content and value come from. Infrastructure is most valuable when it has disappeared and “just works,” but how do we remind users that information infrastructure has a cost and requires effort? Publishers are concerned about sites that facilitate discovery of their content but then drive traffic away from the publishers’ sites. Publishers are also concerned about sites that may change DOI or citation information to benefit themselves.

A user may choose to access content through a pirate site due to its ease of use; unawareness that their library has the content immediately available; a need for content for text or data mining; a lack of arbitrary road blocks on numbers of merited downloads, devices, or data sizes; and speed (as proxy servers are network-bottlenecked, which users notice).

#### Group 2

Group 2 considered pirate sites to include ResearchGate and SciHub, and maybe even Mendeley and Endnote as they permit file sharing. However, the difference between SciHub other file-sharing platforms is that SciHub actively breaks down barriers with criminal or at least malicious intent by subverting authentication procedures and downloading content *en masse*. The use of SciHub may be greater in larger research institutions. Although universities exert consequences for file sharing, they take no steps against article sharing.

Value added by publishers includes the provision of datasets, promotion of articles, and presence of impact factors. Unbundling these services would probably not be effective. Also, a problem with green access and preprint servers is that there is no functionality to definitively indicate whether an article has been accepted or rejected by a journal.

#### Group 3

Group 3 believed that the benefits of pirate sites were their ease of use and quick results. Regarding preprint servers, however, we worried about the quality of the information: Is a preprint the best version?

We considered some solutions to the problem of difficult access to scholarly information, such as reestablishing the academic system to reward open access publishing; creating a tool that provides a workflow for accessing a PDF through potential sources, directing users to purchase access if it is not freely available; and developing a notification system to alert libraries to broken link resolvers.

### Security and the future of IP authentication

#### Questions to consider

- What are the most important ways in which IP authentication/proxy servers fall short of current needs?
- What are the most complex and/or time-consuming pain points in terms of administering IP authentication and proxy servers for adminstrators at both libraries and publishing organizations?
- What are the scenarios that keep publishers and librarians up at night in terms of security of licensed information and user privacy?
- Knowing what we now know about IP authentication, what are the major pitfalls to avoid in the implementation of a new authentication regime?

#### Group 4

Group 4 considered an experience common to libraries: A user’s credentials are compromised, and a vendor notices excessive downloading and requires the library to address the problem or risk loss of access. Librarians recognized this work as routine, noting the effort spent on managing proxy-based systems. Librarians considered this as labor expected by the vendor community. Publishers have compromised their control of content when so much has been uploaded to pirate sites: “What do we do when the proverbial horse has left the barn?”

Are we spending too much effort worrying about bad actors? Should we instead consider ways to enable and facilitate access by users? Our group had consensus about the “wrong-headedness” of blocking access as retribution, noting “it takes little to compromise lots.”

Some publishers are creating ecosystems to engage authors “from cradle to grave,” with the hope of forming “sticky” partnerships with future content-generators and, thereby, establishing lifelong brand loyalty. Does the commodification of researchers lead to the diminution of diverse perspectives in the marketplace? How can we preserve a diversity of publisher voices in light of scaled operations? What is the impact on small or specialized publishers? What happens to dissonant or marginalized voices?

Some publishers in our group admitted that they feared the consolidation of libraries, publishers, and health care organizations, noting “scaled solutions are abetted by the digital revolution.”

We also questioned what digital literacy skills are reasonably expected of content users. Do we expect them to understand copyright? To avoid compromising their credentials? To practice good privacy and digital hygiene?

#### Group 5

Group 5 identified several problems with IP-based authentication. Changes in IP addresses must be distributed to numerous vendors, which is time-consuming for all. Although tools are now available to help with this distribution, they are “just Band-Aids.” Hospital firewalls often make offsite downloading difficult. Proxy issues occur when a publisher changes to a secure HTTP, which is a problem on both sides, as the publisher cannot provide the technical support needed to change proxy settings for the library, and the library may not have sufficient institutional technical support. Also, a single IP range can cover multiple locations, but a product license does not. Although multifactor authentication has been recommended for security, this is just another barrier to content.

Libraries and their technical staff are not able to adopt new authentication standards very quickly, leaving vendors to support many different systems and standards, which compromises their ability to improve a product’s user experience. Libraries get frustrated because a product “doesn’t work,” although the problem may be out of the vendor’s control. Therefore, it is important to bring librarians into discussions on new authentication methods.

Overall, our group felt that a user’s ease of access is more important than our collection of data.

#### Group 6

A hospital librarian in Group 6 worried about users being unable to access content: “the hospital takes care of security, but it is restrictive.” Some users download a single copy from a vendor and store it on a local network for others to access, which prevents accurate usage statistics.

We referred back to Garrity’s talk when he said, “experience what the user experiences when finding content.” When people access content through the library and proxy server, is this a good experience? Trace the pathway from the library website to the publisher website; once a user arrives at the publisher’s site, they may still have difficulty finding a PDF due to variations in publisher interfaces.

Library patrons with legitimate access to content have too many hoops to jump through. There are too many authentication options on some sites. Also, a system that asks for Shibboleth or OpenAthens authentication is using jargon and talking “insider baseball” (a detail-oriented approach to the minutiae of a subject). Users do not care where they get content as long as they get it.

A wild idea is to open all geographically defined access to a specific article based on IP addresses in a city like Birmingham, AL, or Lincoln, NE, when it is needed for a journal club or similar purpose. If a user geolocates to a licensed major institution area, then they should be able to access the resource. However, this would not work in a bigger city with several major medical schools in a small area. Also, firewalls sometimes block location access in hospital settings.

RA21 relies on a third party to verify user identity, and this third party must be trusted on all sides. A federated identity could solve some access problems, but a trusted identity provider is required.

In summary, we must create a hassle-free experience for users.

## KEYNOTE #2: ARTIFICIAL INTELLIGENCE AND ENHANCED SEARCH

### Ruth Pickering, Co-Founder and Chief Strategy and Business Development Officer, Yewno, Redwood City, CA

Most searchers confine their discovery to the first page of Google results, without digging any deeper. In her keynote lecture, Ruth Pickering described a new approach to making search results more meaningful and adaptable to a user’s specific needs.

Pickering first described the history of a new idea for information discovery. Born out of research in the field of applied mathematics, initially in the field of econophysics focusing on the behavior of financial markets and later in biomedical research identifying potential drug targets for rare diseases through mining millions of journal articles, librarians at Stanford University proposed that a similar approach could be used as an interdisciplinary discovery tool. That is, a technology capable of ingesting and analyzing massive amounts of unstructured information could break down disciplinary silos by allowing users to query one place and get results that span disciplines. The resulting product was Yewno.

The proof-of-concept of Yewno was demonstrated at Stanford University in 2015 with 10 million pieces of content (i.e., Wikipedia and items from the university’s standard collection). It then moved into beta testing at several institutions of varying sizes, which showed that 90% of undergraduate students found a new connection between concepts in an already familiar research area. At its launch in 2016, Yewno used its content of 45 million items to generate graphs visualizing connections between concepts. As its content continued to increase, however, these graphs became more complicated. Thus, the new version of Yewno presents clearer graphs and more structured results, with further refinements expected in the future.

Yewno works using artificial intelligence (AI). Computer algorithms are applied to a huge volume of digital information from government, scholarly, business, and news reports to create human-like inferences. Meaning is extracted from the information through the identification of keywords in their context to form unique concepts. Relationships among concepts are represented as connections with connection weights, as in a neural network model. In this manner, users can see not only that connections exist, but also *why* the connections exist. Using a blend of computational semantics, graph theory, and machine learning, Yewno complements databases by recreating how the human brain works on an enormous scale.

Pickering reminded participants that AI is a huge field expected to be main driver of productivity and economic growth for at least the next twenty years. Although AI is already here (e.g., personalized recommendations by Amazon and Netflix, voice-based virtual assistants like Siri), it is still in its beginning stages, and we must be prepared for further change. Pickering has heard a range of publisher reactions to AI; most are excited, although some express concern. However, AI is not about replacing people but about helping people make better decisions.

Pickering demonstrated Yewno in use. One example was a search for “autism” and “MMR vaccine.” The search results included a text-based overview of the topic, definitions of concepts, and links to relevant documents and featured a graph of connections between the concepts “autism,” “MMR vaccine,” and “controversies in autism.” Some of these connections were expected, whereas others were surprising. Pickering showed that Yewno does not provide a definitive “answer”; rather, it is a research tool that helps users sift through massive amounts of literature. Using Yewno is an engaging experience; users can click on anything, which encourages active search and tailors each exploration to a user’s unique research needs.

Pickering also demonstrated Yewno Life Sciences, a separate tool for expert users that additionally contains clinical trial and genetic information. She first searched for “Middle East respiratory syndrome,” for which there is no approved drug. When she entered the keyword “dipeptidyl peptidase 4,” the diabetes drug sitagliptin that targets the dipeptidyl peptidase 4 protein appeared in the graph as a connected concept, with accompanying information on relevant drug trials, targets, and interactions. Thus, Yewno Life Sciences can help researchers make connections between diseases and drug targets in biological systems.

Finally, Pickering showed how Yewno can provide metrics on user engagement. Product administrators can see actions performed by users such as viewing snippets (i.e., keywords embedded in their context), adding or removing concepts, reading articles, and sharing their results. They can also see which concepts, topics, and disciplines are most highly explored and which documents from various publishers are most frequently accessed, which can be valuable information for specific institutions and user communities.

### Question-and-answer session

Several questions from the audience concerned basic information about Yewno, such as its coverage, mechanics, and mode of institutional access. In response, Pickering explained how Yewno receives full-text content from publishers and then directs traffic back to publisher sites through links to articles. Yewno also ingests content from preprint servers and many conference proceedings. Users can filter Yewno search results to show only content available through their institutions. Yewno is a subscription-based product based on FTE ranges, and subscribers are provided with data on their user engagement.

Other questions reflected audience members’ concerns about how Yewno might influence the information presented to users and, thus, perpetuate bias. A librarian questioned the degree to which the company mediates the choice of content ingested into Yewno. Pickering assured the audience that Yewno is neutral and unbiased; they do not own or sell any content, and they expose all connections ranked by their strength rather than by which publisher supplied the content. Another participant questioned the responsibility of the company in selecting content, such as possibly deciding not to include “false literature” on the autism-vaccine link. Pickering stated that they trust the publishers that provide content to Yewno and do not censor the literature. Yewno does not “enter the debate” but only surfaces links, which must ultimately be evaluated by the user. A librarian supported this stance by saying that a sufficiently large body of content would serve to place controversies (e.g., the autism-vaccine link) into perspective.

Another librarian asked whether the age of the content was considered by Yewno algorithms, wondering whether it was good if outdated arguments resurface (e.g., eugenics). Pickering said that users can filter search results based on the age of the content but noted that she needed to investigate how the algorithms treat older versus newer content. A publisher asked whether the semantics used by Yewno were dynamic, noting that definitions of terms change over time. Pickering explained that concepts are adaptive unique entities and that Yewno defaults to the most recent terminology.

A final set of questions addressed whether Yewno might be too difficult or too easy to use. A librarian thought that students might not use Yewno because it is not easy enough. Pickering countered by stating that they spent a lot of time creating a tutorial, but it ended up being little used. She has observed that students are happy to use Yewno and “click around.” Because users are “in charge” of their discovery, Yewno is perceived as not only easy to use, but also interactive and fun. However, another audience member felt that Yewno might be too easy to use and might serve to take the intellectual work out of research, thus sidestepping reading and true understanding. Pickering stated that users still have to “do the work” to understand the search results. Rather, Yewno “takes away challenges related to the volume, fragmentation, and reputation of content” and helps users find what they are looking for more quickly, allowing more time to reason.

## PANEL #2: CHALLENGES IN DEVELOPING INFORMATION LITERACY IN THE MEDICAL CENTER

The objective of this panel was to share approaches to teaching users how to determine the value and credibility of different kinds of information resources. Participants were expected to become more adept at demonstrating the value of thoughtfully curated information products and collections.

### Challenges in promoting information literacy in an academic medical center

#### Shalu Gillum, AHIP, Head of Public Services, Harriet F. Ginsburg Health Sciences Library, College of Medicine. University of Central Florida–Orlando

Shalu Gillum described her library as a success story of user engagement through its focus on teaching IL to both faculty and students. Her library opened in 2009 as a “born-digital” library with the motto “Information, Anywhere, Anytime, on Any Device.”

Gillum said that the primary IL challenge facing faculty is predatory journals that lure in authors who are desperate to publish in order to achieve promotion or tenure. Librarians’ solution is to deliver brief presentations on predatory publishing in departmental faculty meetings, teaching faculty how to distinguish between legitimate and illegitimate journals and seeking to dispel the myth that open access publishing is equivalent to predatory publishing. Their goal is to help faculty see librarians as partners in the publishing process and to give them tools for evaluating journals (e.g., Editage Insights checklist, Allen Press’s phony versus legit infographic) rather than relying on a blacklist. As a result, librarians have received positive emails from faculty, some boasting about their ability to identify email solicitations from predatory journals.

The primary IL challenge facing medical students is their use of non-authoritative (e.g., Google) or inappropriate (e.g., consumer health websites) resources to answer clinical questions. Librarians’ solution is to integrate into the Practice of Medicine 1 course and provide feedback on students’ use of resources to answer patient-based questions. Librarians introduce students to a LibGuide with links to evidence-based medicine (EBM) resources and provide feedback to students via LiveText on their monthly clinical question assignments using a rubric codesigned with faculty. Their goal is to train students to use EBM resources when answering clinical questions. As a result, they have seen improvements in students citing and providing links to appropriate EBM resources (e.g., UpToDate, DynaMed).

Gillum believes that her libraries’ success with faculty depends on developing long-term librarian-faculty relationships, which works due to her institution’s small size and the faculty status of librarians. These librarian-faculty relationships have helped librarians be seen as information experts and have catalyzed their integration into the curriculum, which, along with the Personal Librarian Program, have led to student success in IL.

### Challenges and opportunities in a paradigm shift

#### Amanda DiFeterici, Senior Manager of Product Strategy, Credo

Amanda DiFeterici described a recent shift in higher education toward emphasizing the teaching and assessment of skills that students need for future success in the workforce and posited that these skills could include the foundational skills of IL and critical thinking. Although this paradigm shift poses challenges to librarians, with the right approach, these challenges can be transformed into opportunities.

One challenge faced by librarians is a structural challenge. Whereas the teaching of IL is often front-loaded into the beginning of a semester or program using a “one-shot” approach, IL is a skill that is built and mastered over time. Although students are being asked to perform more complex research, this is not being matched with appropriate IL instruction. Furthermore, there is neither sufficient time nor enough librarians to teach IL in all classes. Another challenge pertains to faculty perceptions of IL and librarians. Faculty tend to view IL as a “checkbox,” whereas IL should be integrated throughout the curriculum. As faculty are seen as experts, it may be difficult for them to accept that librarians also have expertise, and teaching often falls to the bottom of their priority list en route to tenure.

DiFeterici asserted that student exposure to IL should be relevant, authentic, continuous, and increasing in complexity. It should be an upward spiral, with students revisiting IL skills throughout their coursework and programs. To realize this possibility, DiFeterici made several recommendations to librarians.

First, get explicit about defining and teaching IL. IL should be redefined as a high-value skill set with an impact on lifelong learning and career success. IL learning outcomes should be measurable, and instruction should employ backward design. Also, students should have an awareness of what IL skills are and how these skills will be assessed.

Second, develop IL skills over time. As one-shot instruction is ineffective, librarians should work toward an embedded model in which they go into classes repeatedly and help scaffold IL skills into courses or programs. For example, a big assignment can be broken into smaller steps, each aligned to an IL learning outcome. The focus should be on the process rather than the product.

Third, teach IL by “doing.” Lectures should be replaced by active learning activities. For example, in-class time could be used to help students dig into databases, whereas out-of-class time could be supplemented with videos and tutorials. Tie skills into “real-world” projects that focus less on accumulating knowledge and more on acquiring skills.

Fourth, reframe the value of the library. Relating IL to critical thinking may help gain traction with faculty and campus administrators. Demonstrate how librarian roles are shifting from managing collections and being gatekeepers of information to being co-teachers who collaborate with faculty to design classes, deliver instruction, and deploy online learning systems.

DiFeterici concluded by proposing that librarians can overcome challenges by embracing the “M-word” (i.e., marketing). Gather evidence that libraries improve student success and develop an elevator pitch for “Why IL? Why the library?” Get on a committee—any committee that gives you a connection to others concerned with student success. Identify friendly faculty who will work with you to try embedded librarianship and allow “evangelist faculty” to spread the word to their peers. Make friends with staff in institutional assessment offices, and start tracking grade point averages and graduation data from students who receive IL instruction. Communicate your value at every opportunity—not only the resources available in your library, but also your expertise.

### The social life of health information, its importance to user engagement, and its implications for information literacy

#### Kelsey Rosell, Vice President of Institutional Sales, Digital Science

Kelsey Rosell spoke about the advantages of altmetrics over traditional metrics for assessing research impact in an age in which research is disseminated and discussed more broadly than ever before.

The technology revolutions of broadband Internet, smartphones, and social media/networks have changed how and where people access information as well as the boundaries between people and information. Engagement with reports of health sciences research now takes place on a variety of online platforms for different user populations, including reporting and information-sharing platforms (e.g., news media, Wikipedia, blogs), commentary platforms (e.g., Reddit, peer-review sites, comment threads), social media platforms (e.g., Facebook, Twitter, LinkedIn), and practitioner platforms (e.g., Doctor Evidence, Medmeme, Doximity). Health sciences research is now read not only by other researchers, but also by practitioners (e.g., health care providers, lawyers, legislators, teachers), governmental and nongovernmental agencies, interested parties (e.g., patients and family members, community groups, advocates), and the general public.

The Internet is now the most utilized information resource for both health care providers and patients. The vast majority (86%) of physicians use the Internet to gather medical and pharmaceutical information, with fewer relying on resources such as online continuing medical education (CME) courses, peer-reviewed journal articles, pharmaceutical sales representatives, colleagues, and books.

As an example, Rosell told the story of a doctor with a large social media following who published a journal article on sepsis in early 2018. Posting about his article on Facebook attracted the attention of his social media followers and set off a chain reaction of online public engagement. Altmetric data showed that his article was tweeted over 800 times and was picked up by news media in different countries, leading to his treatment recommendations being put into practice. To date, however, his article has received only one traditional citation. This case exemplifies how altmetrics can be more revealing of the impact of scholarly research than traditional impact metrics.

Rosell also provided examples of how technology revolutions are changing communication between physicians and patients. Some doctors send emails to their patients with information about new treatments and recommended reading for further understanding their medical conditions. Other doctors may share information from journal articles with their patients on social media platforms. Such interactions with the medical literature by physicians and patients are likely not captured by traditional impact metrics.

In conclusion, Rosell asked librarians to think about their role in promoting research engagement and measuring research impact by asking themselves questions such as: How does my medical school currently define impact? Which platforms and metrics best support our values and vision? How might these platforms and metrics vary by discipline? What audiences do we want to reach? How can we help researchers and health care practitioners increase audience engagement with their work? How can we incorporate social collaboration networks and altmetrics into our existing institutional workflows and reporting processes?

### Question-and-answer session

A major topic of audience discussion was the absence of expertise in public discussions of health-related topics and the responsibility of publishers and librarians in countering misinformation and improving IL skills. A publisher expressed fear of the growth of casual comment on social media on topics such as vaccinations, stating that lay people debating medical evidence is “more of a concern than something to be celebrated.” Rosell responded, “I agree. It’s scary, but it’s there,” noting that authors have to engage with social media to communicate their research. She said this is why it is important to understand the platforms through which researchers disseminate their work, hinting that publishers and librarians should also be active on these platforms.

Another participant proposed that IL training should encompass social media, such as how authors can responsibly counter misleading or harmful tweets about their work. A librarian stated that libraries have to make the connection between IL and digital literacy by using tools such as the Association of College & Research Libraries’ Framework for Information Literacy for Higher Education to provide “a broader lens through which to look at information sources. DiFeterici agreed that IL is “not just about how to search and how to cite,” and Gillum expressed hope that librarians can bring IL into patient education by teaching medical students how to talk about EBM with patients who bring in information from the Internet.

Another topic of discussion was the need for publishers and librarians to work together to develop resources for authors for promoting their work online and using altmetrics. A participant asked how publishers educate authors about using social media to attract an audience for their work. A publisher replied that they use several strategies (e.g., plain-speak summaries, Kudos) to make scholarly work approachable from all levels and provide authors with information packets on the benefits of promoting their work. Another publisher asked how they could help libraries in this area. A librarian responded that publishers could create “small chunks” of materials (e.g., brief videos and screenshots) with copyright licenses permitting reuse that could be embedded in LibGuides or library tutorials. This librarian also emphasized the need to incorporate author services or education into the research workflow. The session ended with the idea that libraries and publishers could cocreate curricula for authors.

## SMALL GROUP EXERCISE #3

Participants gathered in small groups with roughly equal representation by librarians and publishers to discuss the topics of “leveraging specialized discovery tools to maximize user engagement” and “imagining the ideal social networking site for collaboration and sharing.” The groups defined broad problems, identified areas of concern or controversy, and suggested next steps.

### Leveraging specialized discovery tools to maximize user engagement

#### Questions to consider

- What types of information are available through the library but invisible through standard library automation systems? Is there a need to build tools to search through this information?
- Do existing discovery tools return information in a form that is typically useful to the searcher? How could the search returns be better organized?
- What would be the value to the user of federating search results from many discovery tools?
- To what degree would an optimal searching environment enhance the satisfaction and engagement of existing users? Could such an environment encourage user loyalty to a particular platform/publisher? Could such an environment serve to attract potential patrons who are not currently engaged with the library?

#### Group 1

Although our group questioned the need for another discovery system when we already have tools like Primo, Summon, and EBSCO, we thought that an ideal discovery tool should be like Google in that it searches for articles, books, images, videos, and other types of content at the same time. We thought data visualization should also be incorporated, as it has the “cool” factor.

However, if all publishers cannot work together on a discovery tool, then there is still a problem in discovery. For a universal discovery system to work, publishers must agree to adopt some form of universal indexing. Publishers in our group said they do not want to restrict discovery of their content, but indexing their information remains a challenge.

An important consideration is the reliability of discovery systems, which tend to reduce results to a lowest common denominator or “just good enough.” While this suffices for undergraduate students writing papers for class, it is not good enough for librarians assisting physicians.

Federated searching has not gained traction in the medical world because the outcomes of searching for medical information are important. Librarians in our group said they must trust a search tool to gather all relevant and important information, as they have been burned in the past by badly designed discovery tools (e.g., WebFeet). A good librarian will use multiple platforms including PubMed and publisher databases to perform a comprehensive search, as patient care can be negatively affected by inaccurate or incomplete results. Thus, an abundance of caution is needed when designing and using discovery tools for medical information.

Finally, if medical students are taught to use a subscription-based search tool, then they are handicapped when they are residents at an institution that does not have access to that tool. This is why librarians often teach expert searching using PubMed, which is accessible to everyone.

#### Group 2

No one in our group was convinced that their institution was using a “specialized discovery tool.” None of us had heard of ScienceOpen or TrendMD, but we were familiar with Yewno (which was considered a “nice to have” rather than a “need to have”). Our closest examples of specialized discovery tools included Dimensions, application programming interfaces (APIs) built on top of discovery platforms, and ResearchGate. We were not completely convinced that specialized discovery tools were right for all users or would make searching easier for all users.

We believed that the value of discovery tools to the user comes from saving time, learning about new information resources, having the ability to filter results, and having the ability to identify gaps in research that could be fruitful areas for future publication.

In an ideal world, discovery tools would have snippets (i.e., terms appearing in context), distinguish between items with immediate versus delayed access, provide easy links for requesting interlibrary loans, allow library branding, have link-outs embedded in normal workflows (e.g., learning management systems, electronic medical records), and direct users to librarians if they need further assistance rather than leading to dead ends in their search.

Finally, we did not think a “specialized discovery tool” would attract patrons to the library’s website if they were not already library users.

#### Group 3

Whether a user discovers information using PubMed, Scopus, Summons, Primo, or a clinical diagnostic tool, how the search results are organized should ultimately depend on what best suits the user. The question is how to “curate” the discovered content in a way that is meaningful to the user’s search topic. The search results interface should also map to a subject area librarian who has the expertise to guide the user along another discovery path if the search results do not meet the user’s expectations. There should be no dead-ends: users should be presented with a next step (e.g., contact a human expert to explore other search strategies, training opportunities, or different discovery tools). Furthermore, patterns in discovery tool input error could be identified to allow automatic correction by the system.

We believe that librarians are in a unique position to provide training on discovery tools and teach related concepts like critical appraisal to enhance the search and discovery process.

In summary, search is only one component of the discovery process. Librarians should have ongoing conversations with user communities to elucidate desired features in discovery tools. Armed with this information, librarians can work closely with vendors to request and beta-test enhancements in information resources.

### Imagining the ideal social networking site for collaboration and sharing

#### Questions to consider

- For health sciences users, what are the major workflows involving shared data, writing, etc.?
- To what degree do users employ, or at least know about, generalized collaboration platforms (e.g., Slack)?
- What specialized collaboration platforms do your users currently mention? What is attractive about their functionality?
- What is the place on an ideal site for use of licensed content?
- Should such a platform be hosted and administered by the library, by academic programs, by a research office, or by a publisher? Why?

#### Group 4

We considered collaboration and sharing as including (1) the sharing of data and code, (2) ongoing work collaboration, and (3) internal and external engagement with the academic/medical community and beyond. These are widely different facets that cannot be fulfilled by any single social networking site. Instead, it may be better to maintain separate tools serving distinct functions with the possibility of their automated integration.

Tools serving these three components of collaboration and sharing are already in widespread use, and our group favored the institutional adoption of appropriate popular tools to optimize participation. We thought the role of libraries is to facilitate, mediate, and teach users about these tools, with overall ownership of the endeavor residing elsewhere in the institution. Tool selection should be based on their compliance with mandates and laws (e.g., Health Insurance Portability and Accountability Act [HIPAA]), security, scalability, and versioning.

Examples of effective existing tools include general collaboration platforms (e.g., Slack, Microsoft Teams, Google Groups), workflow tools (e.g., Mendeley), data and code sharing sites (e.g., Figshare), and community-building platforms (e.g., ResearchGate, F1000, Vivo).

#### Group 5

Our group joked that an ideal social networking site for collaboration and sharing would be a “Facebook for scientists.” Member of our group were familiar with Slack, collaborative platforms for sharing documents, and the use of tools such as Browzine and Twitter to find and discuss journal club articles. However, hospital librarians in our group indicated that many social media sites and chat tools are blocked by firewalls due to HIPAA concerns. Some librarians suggested that publishers could authorize temporary access to full-text articles to facilitate access for journal clubs, which could help avoid copyright issues.

We did not see a need to develop a new single networking tool, as people have different approaches and work in different environments. Whatever is embedded into the institutional fabric is what users will use, and perhaps the time for a single tool has already passed.

#### Group 6

Collaborative networks connect disparate scholars/researchers with each another, which increases the efficiency of science and discovery by reducing duplicate efforts and enabling synergistic partnerships. We believed an ideal social networking platform would necessarily allow the sharing of content among scholars, but this sharing would be a means to an end (i.e., leading to meaningful collaboration) and not its main objective.

Our group envisioned a platform that provided a “Match.com-like” way for researchers and scholars to find each other and explore mutual interests. It would provide collaborative workspace for groups that wanted to pursue projects together, similar to F1000Workspace. It would also assist in creating a “submittable” manuscript and allow sharing of the final product (i.e., a published paper or conference abstract).

An ideal platform would have the following characteristics:

- Broadly defined scholarly membership: Silos of research are not conducive to new discovery; there must be structures that encourage multidisciplinary interaction and serendipity. A successful outcome would be an increase in the number and diversity of authors on papers.
- Interoperability with existing systems: New platforms that increase the administrative burden of university and hospital information professionals will not work. Any workflow inputs, outputs, and link-outs must work with currently employed systems and platforms.
- Author services: Incorporation of preprint (i.e., manuscript preparation) and postpublication (i.e., altmetrics) author services.
- Legitimate sharing functions: An ideal platform would solve some problems of illegitimate file sharing by incorporating the sharing of links in accordance with RA21 and STM guidelines.
- Governance with mutual confidence: All stakeholders must have trust in the governance structure, such as reliance on an organization like ORCID.

## PLANNING SUMMIT OUTCOMES

### Key questions

In the final session, participants collectively identified key questions formed during the summit that were expected to impact their work or their organizations’ strategies and that could stimulate the creation of enduring materials to advance the causes of user engagement and medical libraries.

#### Disrupters

- What should we do about ResearchGate?
- What will happen with medRxiv?
- How do we make it easier or more desirable for users to employ legitimate means of access to combat piracy?

#### User focus

- How can we embed social media/article sharing into discovery services? Could publishers provide authors with best practices for marketing and sharing their work?
- How can we better understand our users’ needs and integrate our services into their workflows? How can we better understand the drivers of change in user needs?
- How can we better understand how our discovery tools are being used and assess whether we are returning the most needed content (as opposed to all content)?
- Should we survey users on their searching habits and preferred use of discovery tools?
- How can we be more mindful of our users, so that our value outweighs our annoyances?

#### Authentication

- How can we speed up the process of finding solutions to problems of IP authentication?
- What is next for authentication? Is there a clear winner? Are we prepared for a fragmented market?
- In anticipation of changes in authentication methods (e.g., RA21), how should libraries and publishers start preparing?
- How should the publishing industry and libraries deal with the threats of no more IP authentication and everything being open access?

#### Information literacy

- How do we integrate IL into user workflows, including their use of scholarly communication networks?
- What can publishers do to help librarians with IL training?
- Do librarians and publishers have a shared responsibility to educate users on authority and misinformation? Is it time to cocreate digital literacy programs?
- How can we create tools that facilitate information discovery while also requiring users to employ IL and critical thinking skills?

#### Common ground and future positioning

- How can we position ourselves to maintain relevance in the future when more information is freely available?
- Do librarians and publishers have more in common than differences in terms (e.g., shared values)?
- How can librarians and publishers move beyond negotiation to collaboration?
- Can publishers and librarians cocreate digital literacy programs and author guides on content sharing, copyright, and related issues?
- How can we increase our focus on assessment and analytics from both library and publisher sides?
- How can we ensure additional summits and continued discussion and outlets for larger conversations?

### Potential outputs

After the summit, the program committee made the following suggestions for tangible summit outputs that librarians and publishers could cocreate:

- Panel presentation during the MLA InSight Initiative Summit 1 Outcomes Open Forum at MLA ’18
- Publications memorializing key summit takeaways (e.g., commentaries in the *Journal of the Medical Library Association*, Scholarly Kitchen blog posts)
- Joint statements or white papers on specific issues (e.g., sharing and promoting one’s work, perpetuation of bias by discovery systems)
- Practical information resources (e.g., LibGuides)
- Digital literacy curriculum for authors
- In-person or online continuing education courses for MLA members

## PRE-SUMMIT PARTICIPANT READING LIST

### Leveraging specialized discovery tools to maximize user engagement

Conrad LY. The latest in search: new services in the content delivery marketplace. The Scholarly Kitchen [Internet]. 2 Nov 2016 [cited 22 Feb 2018].
  <https://scholarlykitchen.sspnet.org/2016/11/02/the-latest-in-search-new-services-in-the-content-discovery-marketplace/>.
Hinchliffe LJ. Discovery should be delivery: user-centric principles for discovery as a service. The Scholarly Kitchen [Internet]. 8 Jan 2018 [cited 22 Feb 2018].
  <https://scholarlykitchen.sspnet.org/2018/01/08/discovery-delivery-user-centric-principles-discovery-service/>.
Hanneke R, O’Brien KK. Comparison of three web-scale discovery services for health sciences research. J Med Libr Assoc. 2016 Apr;104(2):109–17. DOI: http://dx.doi.org/10.3163/1536-5050.104.2.004.

### Security and the future of IP authentication

Michael A. Ask the chefs: where is the balance between security, authentication, marketing, and privacy? The Scholarly Kitchen [Internet]. 1 Dec 2016 [cited 22 Feb 2018].
  <https://scholarlykitchen.sspnet.org/2016/12/01/ask-the-chefs-where-is-the-balance-between-security-authentication-marketing-and-privacy/>.
Popowich S. Beyond IP authentication in libraries [Internet]. 23 Nov 2017 [cited 22 Feb 2018].
  <http://redlibrarian.github.io/article/2017/11/23/beyond-ip-authentication.html>.

### Lessons learned from pirate sites

Graber-Stiehl I. Science’s pirate queen. The Verge [Internet]. 8 Feb 2018 [cited 22 Feb 2018].
  <https://www.theverge.com/platform/amp/2018/2/8/16985666/alexandra-elbakyan-sci-hub-open-access-science-papers-lawsuit>.
Green T. We’ve failed: pirate black open access is trumping green and gold and we must change our approach. Learn Publ. 2017 Oct;30(4):325–9. DOI: https://dx.doi.org/10.1002/leap.1116.
Pelcastre IF, Correa FG. The current system of knowledge dissemination isn’t working and Sci-Hub is merely a symptom of the problem [Internet]. London School of Economics and Political Science; c2016 [cited 22 Feb 2018].
  <http://eprints.lse.ac.uk/70381/1/blogs.lse.ac.uk-The%20current%20system%20of%20knowledge%20dissemination%20isnt%20working%20and%20Sci-Hub%20is%20merely%20a%20symptom%20of%20the%20pro.pdf>.

### Imagining the ideal social networking site for collaboration and sharing

DeLory C. Scholarly communication issues around scholarly collaboration networks. Libr Connect [Internet]. 2017 Oct 25 [cited 22 Feb 2018].
  <https://libraryconnect.elsevier.com/articles/scholarly-communication-issues-around-scholarly-collaboration-networks>.
Rapple C. Updated figures on the scale and nature of researchers’ use of scholarly collaboration networks. The Scholarly Kitchen [Internet]. 7 Apr 2017 [cited 22 Feb 2018].
  <https://scholarlykitchen.sspnet.org/2017/04/07/updated-figures-scale-nature-researchers-use-scholarly-collaboration-networks/>.
Staniland M. How do researchers use social media and scholarly collaboration networks (SCNs)? Nature.com community blog [Internet]. 2017 Jun 15 [cited 22 Feb 2018].
  <http://www.springersource.com/scholarly-collaboration-networks/>.

## MLA INSIGHT INITIATIVE TASK FORCE

The task force is the steering committee for the multi-year InSight Initiative. The task force also reviews the applications from librarians expressing an interest to attend an InSight Summit and selects the participants based on the summit theme and a representative mix of librarians affiliated with the diverse organizations with whom vendors work, including academic medical centers, community hospitals, specialty schools (nursing, pharmacy, etc.), governmental agencies, corporations, and nonprofit advocacy and community-based organizations.

Gerald J. Perry, AHIP, FMLA, Chair, University of Arizona, MLA Past President
Barbara A. Epstein, AHIP, FMLA, Member, University of Pittsburgh, MLA President
Michelle Kraft, AHIP, Member, Cleveland Clinic Foundation, Member, MLA Past President
Gabriel R. Rios, Member, Indiana University, Member
Daniel J. Doody, Summit Organizer, Doody Consulting
Rich Lampert, Summit Co-Organizer, Doody Consulting
Beverly Murphy, AHIP, FMLA, Board Liaison, Duke University Medical Center, MLA President-Elect
Kevin Baliozian, Member, Medical Library Association, MLA Executive Director
Mary M. Langman, Staff Liaison, Medical Library Association

## INSIGHT SUMMIT 1 PROGRAM COMMITTEE

The program committee developed the schedule and all program elements for InSight Summit 1. It was appointed by the InSight Initiative Task Force and consisted of three librarians, three representatives from the participating organizations, the program facilitators, and a liaison from the InSight Initiative Task Force.

Caitlin Cricco, Member, Industry Representative, Springer Nature
Nadine Dexter, AHIP, Member, Library Representative, AAHSL Board Representative, University of Central Florida
Richard Gallagher, Member, Industry Representative, Annual Reviews
Donna Gibson, Member, Librarian Representative, Memorial Sloan Kettering Cancer Center
Linné Girouard, AHIP, Member, Librarian Representative, Houston Methodist
Steven Heffner, Member, Industry Representative, Wolters Kluwer
Daniel J. Doody, Summit Organizer, Doody Consulting
Rich Lampert, Summit Co-Organizer, Doody Consulting
Gabriel R. Rios, Liaison, InSight Initiative Task Force, Indiana University
Mary M. Langman, Staff Liaison, Medical Library Association

## INSIGHT SUMMIT 1 FACILITATORS

Discussions and group exercises were facilitated by InSight Summit Program Committee members.

Daniel J. Doody, Doody Consulting
Rich Lampert, Doody Consulting
Gabriel R. Rios, Indiana University

## INSIGHT SUMMIT 1 PARTICIPATING ORGANIZATIONS AND SPONSORS

MLA thanks the following participating organizations:

Annual Reviews
American Psychiatric Association Publishing
BMJ Publishing
Elsevier
F1000
The JAMA Network
McGraw-Hill Education
NEJM Group
Springer Nature
Wolters Kluwer

MLA also thanks the Association of Academic Health Sciences Libraries (AAHSL) and Elsevier for their financial support of the travel expenses and registration of librarian participants.

## INSIGHT SUMMIT 1 PARTICIPANTS

The InSight Summit had an equal representation of librarian leaders and participating organizations.

Katherine G. Akers, Wayne State University
Donna R. Berryman, AHIP, University at Buffalo
Harold Bright VI, AHIP, A.T. Still University of Health Sciences
Daniel Burgard, University of North Texas Health Science Center
Caitlin Cricco, Springer Nature
Vida Damijonaitis, JAMA Network
Nadine Dexter, AHIP, University of Central Florida
Richard Gallagher, Annual Reviews
William Garrity, University of California–Davis
Shalu Gillum, AHIP, University of Central Florida
Susan Haering, Massachusetts Medical Society/NEJM Group
Rebecca Harrington, AHIP, Florida State University
Emma Cryer Heet, AHIP, Duke University
Steven Heffner, Wolters Kluwer
Lauren Jones, BMJ Publishing
Elizabeth Laera, AHIP, Princeton Baptist Medical Center
Susan Lamprey, Wolters Kluwer Health
Andrea Lopez, Annual Reviews
Elizabeth R. Lorbeer, AHIP, Western Michigan University
Jason Mathis, Elsevier
Robert McKinney, Massachusetts Medical Society/NEJM Group
Roxann W. Mouratidis, AHIP, Florida State University
Nathan A. Norris, AHIP, Beth Israel Deaconess Medical Center
Gerald J. Perry, AHIP, FMLA, University of Arizona
Steve Quinlivan, Elsevier
Rene Schoelzel, F1000
Frank Scutaro, McGraw-Hill Education
Jonathan P. Shank, Northwestern University
Christine Sontag, Springer Nature
Ryan Steinberg, Standard University
Megan Vance, American Psychiatric Association
Fran Yarger, University of Pittsburgh
Sarah Zimmerman, The JAMA Network

## INSIGHT SUMMIT 1 PRESENTERS

The InSight Summit featured speakers and panelists with expertise on the summit’s thematic topics.

Amanda DiFeterici, Credo
Heather Flanagan, RA21
William Garrity, University of California–Davis
Shalu Gillum, AHIP, University of Central Florida
Lisa Hinchliffe, University of Illinois–Urbana-Champaign
John Inglis, Cold Spring Harbor Press
Ruth Pickering, Yewno
Kelsey Rosell, Digital Science

